# Impact of Multiple Reprocessing on Properties of Polyhydroxybutyrate and Polypropylene

**DOI:** 10.3390/polym15204126

**Published:** 2023-10-18

**Authors:** Priyanka Main, Sandra Petersmann, Nadine Wild, Michael Feuchter, Ivica Duretek, Mariya Edeleva, Peter Ragaert, Ludwig Cardon, Thomas Lucyshyn

**Affiliations:** 1 Polymer Processing, Montanuniversitaet Leoben, Otto-Gloeckel-Straße 2, 8700 Leoben, Austria; ivica.duretek@unileoben.ac.at; 2 Faculty of Engineering and Architecture, Centre for Polymer and Material Technologies, Ghent University, Technologiepark Zwijnaarde 130 (Zone C3), 9052 Zwijnaarde, Belgium; mariya.edeleva@ugent.be (M.E.); ludwig.cardon@ugent.be (L.C.); 3 Faculty of Bioscience Engineering, Ghent University, Coupure Links 653, 9000 Gent, Belgium; peter.ragaert@ugent.be; 4 Materials Science and Testing of Polymers, Montanuniversitaet Leoben, Otto Gloeckel-Straße 2, 8700 Leoben, Austria; s.petersmann@cuas.at (S.P.); nadine.wild@alpla.com (N.W.); michael.feuchter@unileoben.ac.at (M.F.)

**Keywords:** biobased plastics, mechanical recycling, circular economy, polymer processing, PHB, PP, reprocessing

## Abstract

Biobased plastics have the potential to be sustainable, but to explore their circularity further, current end-of-life options need to be broadened. Mechanical recycling is one of the most accepted methods to bring back plastics into the loop. Polyhydroxybutyrates (PHBs) are biobased and biodegradable in nature with promising properties and varied applications in the market. This study focuses on their potential for mechanical recycling by multiple extrusion cycles (E1–E5) and multi-faceted characterization of the virgin (V) and reprocessed materials from E1 to E5. The behavior is compared to polypropylene (PP) as a reference with a similar property profile, which has also been reprocessed five times. The thermal properties of both series showed a stable melting point and thermal decomposition temperature from thermal analyses (differential scanning calorimetry (DSC) and thermogravimetric analysis (TGA)). However, a steady increase in the degree of crystallinity was observed which could counterbalance the decrease in molecular weight due to repeated extrusion measured by gel permeation chromatography and resulted in similar values of tensile strength across the cycles. The strain at break was impacted after the first extrusion, but no significant change was observed thereafter; the same was observed for impact strength. Even in scanning electron microscopy (SEM) images, virgin and E5 samples appeared similar, showing the stability of morphological characteristics. Fourier transform infrared spectroscopy (FTIR) results revealed that no new groups are being formed even on repeated processing. The deviation between the PHB and PP series was more predominant in the melt mass flow rate (MFR) and rheology studies. There was a drastic drop in the MFR values in PHB from virgin to E5, whereas not much difference was observed for PP throughout the cycles. This observation was corroborated by frequency sweeps conducted with the parallel plate method. The viscosity dropped from virgin to E1 and E2, but from E3 to E5 it presented similar values. This was in contrast to PP, where all the samples from virgin to E5 had the same values of viscosity. This paper highlights the possibilities of mechanical recycling of PHB and explains why future work with the addition of virgin material and other additives is an area to be explored.

## 1. Introduction

The European Green Deal [1] and the Circular Economy Initiative [2] as put forward by the European Commission have impacted the life of polymer products, changing it from a linear model (production–use–disposal) to a more circular model (production—use–recycling) [3]. One aspect that needs further evaluation is alternative feedstocks like biobased plastics and their role in the transformation to a circular economy [4,5].

Biopolymers are defined as polymer materials that are biobased, biodegradable or both [6]. Currently, the capacity for biopolymer manufacturing on a global scale is 2.42 million tonnes in 2021, rising to 7.59 million tonnes in 2026. This means that biobased plastics are here to stay and that exploring end-of-life (EoL) options is imperative [7].

Polyhydroxyalkanoates (PHAs) are biobased and biodegradable polymers that are produced as intracellular storage granules by various microorganisms under diverse conditions. PHAs are thermoplastic, biocompatible and non-toxic with good barrier properties [8]. Depending on the number of carbon atoms in the backbone, they are divided into short, medium and long side chains [9,10,11]. Until now, 160 monomers have been identified, and the number is still growing [12,13].

The potential to reduce environmental pollution caused by conventional plastics due to the biodegradability of PHA-based plastics is one of the reasons why they are gaining increased attention. They are also biobased, which means the feedstocks used to produce them are plant-derived sugars or waste streams, making them a promising alternative to fossil-based plastics [14,15,16].

The most studied member of the PHA family is polyhydroxybutyrate (PHB). It has a highly crystalline linear isotactic structure. Additionally, PHB is water-insoluble and relatively resistant to hydrolytic degradation. It has been reported to have a tensile strength of 30–40 MPa and an elongation at break of 2–10%. The melting point of PHB is high at around 177 °C [6,17,18,19].

Recycling becomes a desirable EoL option for the circular economy [3] as the amount of plastic increases [20]. The two main types are mechanical and chemical recycling. Mechanical recycling is the most environmentally attractive and cost-efficient compared to chemical recycling, which needs a lot of new equipment and energy input [21,22]. The different segments of the entire mechanical recycling process consist of collecting, sorting, shredding, washing and final processing to make the recyclates [23].

EoL options for biobased plastics are the same as those for conventional plastics but with some additional options available such as composting. The biobased plastics (bio-polyethylene (bio-PE), bio-polypropylene (bio-PP), bio-polyethylene terephthalate (bio-PET)) are already mechanically recycled along with their petroleum-based counterparts, but for the biobased and biodegradable plastics, the same cannot be said. Technically, biodegradable and biobased plastics can be sorted out by near-infrared spectroscopy, but this is not done yet as the waste streams are very small and the high investment costs are hence not economically feasible. In sorting out plastics, blends of polymers and multilayered plastics are an issue (biobased or conventional plastics). Similar densities of conventional and biobased plastics, e.g., PET and polylactic acid (PLA), introduce additional difficulties to sorting via floatation methods. Having a separate waste stream for these plastics would solve these issues, but currently, no industrial recycling stream for biodegradable and biobased polymers exists [24,25,26]. One of the reasons behind this is the lack of data on the influence of recycling on the molecular and material properties of bio-based polymers.

There have been a few works concentrating on the recyclability of PHAs. The studies range from recycling of the pure polymers PHB, poly(3-hydroxybutyrate-co-3-hydroxyvalerate) PHBV and P(3HB-co-4HB) [27,28,29,30] to recycling via blend design of PLA/PHB, PHBV/PLA, PHBV/poly (butylene adipate-co-terephthalate) PBAT and PP/PHB and the composite synthesis of PHBV/Sisal [31,32,33,34,35,36,37,38]. Since PHBs are expensive and circulate only in small quantities, there are few studies on their mechanical recycling and chemical recycling potential. Even still, the optimal EoL route would be reuse, followed by mechanical recycling and then either pyrolysis or biodegradation. Lamberti et al. [39] state the different EoL options for selected biobased plastics (PLA, PHA, polyglycolic acid (PGA), bio-PET and bio-PE), highlighting once again the importance of recycling method selection based on the analysis of the waste stream. Rivas et al. [27] studied the mechanical recyclability of PHB powder. After three cycles of extrusion and compression molding, a decrease in tensile strength (>50%) was noticed. DSC showed an increase in crystallinity with the number of extrusion cycles which could be due to a decrease in molecular weight. FTIR analysis did not display the formation of any new groups, nor was the thermal stability affected much.

Dedieu et al. [40] reviewed the thermo-mechanical recyclability of biodegradable and biobased plastics with a focus on the degradation mechanisms. With respect to PHAs, the two main pathways of thermal degradation are random β-elimination [41] and/or E1cB elimination reaction proceeding via α-deprotonation [42], both leading to the formation of crotonic acids and oligomers. The degradation mechanism consists initially of a non-auto-catalytic random degradation followed by an auto-accelerated catalytic transesterification process. The self-proliferation of the carboxylic compounds in bulk is needed for the auto-accelerated degradation to begin, and this is caused by unzipping reactions at the ends of the molecules (presumably a zeroth-order kinetic chain scission) [40,43].

In terms of average molar mass, crystallinity, melting point and tensile strength, PHB is similar to polypropylene (PP). Some material properties, e.g., oxygen, fat and odor barrier properties, are superior for PHB than for conventional polymers like PP and poly(ethylene terephthalate) (PET), which broadens the potential for the application of PHB as a sustainable packaging material [18,44,45]. As PHB aims at the substitution of PP for packaging applications, its recycling should be also explored and compared to that of PP.

The degradation mechanism of PP is more dominated by ß-chain scission due to the presence of the methyl group on the backbone which makes it more prone to shearing as occurs during the extrusion process with H-abstraction. This leads to a reduction in molecular weight on repeated extrusion and also increases the degree of crystallinity and hence the Young’s modulus and reduces the elongation at break [46]. Another way for the degradation is due to the attack of oxygen which leads to a reaction with oxygen-based radicals but which is not of import with regard to the extrusion process as the oxygen is consumed in the early stages of processing [46,47]. Canevarolo et al. [48] state that the molecular weight distribution curve shifts away from the original position according to the type and extent of degradation. Upon chain scission, the curve shifts towards the lower molecular weight side. Factors that affect the degradation of PP are the presence of peroxides, screw configuration and processing conditions.

As a lot of work has been conducted on the reprocessing of PP already and only four papers from Alotaibi et al. [49], Hermanova et al. [50] and Tochacek et al. [51,52] dealt with the recycling and characterization of impact copolymer polypropylene (ICPP), we decided to compare PHB with ICPP. Tochacek et al. [47] studied the degradation of ICPP over multiple extrusion cycles and concluded that the degradation behavior is similar to PP as it constitutes around 90% of the mass of the polymer with the effect of the rubbery fraction more pronounced with regard to fracture behavior.

Alotaibi et al. [49] discussed the differences in reprocessing ICPP with a quad screw extruder (QSE) vs. a twin screw extruder (TSE) at three different screw speeds. As the number of cycles progressed and the screw speeds increased, melt temperature had two trends. With the increase in cycles, the melt temperature as well as melt viscosity decreased due to polymer degradation, and less shear heating was produced consequently. With an increase in the speeds, the melt temperature increased due to the high shear generated. As the cycles and speeds increased, the melt mass flow rate (MFR) increased, and the rheological measurements indicated a narrowing of the molecular weight distribution (MWD) and a decrease in weight average molecular weight (Mw). The head pressure, on the other hand, decreased with both increasing speed and number of cycles due to the decrease in melt viscosity for both TSE and QSE. The QSE showed higher melt temperature and greater reductions in molecular weight in comparison to TSE as the three intermeshing zones led to higher shears being generated and showed lower head pressure due to greater free volume present in the QSE. The complex viscosity of reprocessed PP at 500 rpm was similar to that of virgin PP for both screws, but at 1500 rpm, a significant decrease in reprocessed PP was observed for QSE, due to the high shear stresses. Izod impact strength showed a decrease with both increasing speed and number of reprocessing cycles. This behavior was similar in both TSE and QSE.

In this work, we studied the reprocessing of PHB pellets via extrusion and injection molding in comparison to PP as an industrial standard material. We performed five consecutive extrusion cycles for PHB and PP and compared the molecular and material properties; to our knowledge, this is the first time such a comparison has been carried out. We observed no significant influence on the material properties due to reprocessing for PHB, which makes it an attractive option for mechanical recycling.

## 2. Materials and Methods

### 2.1. Materials

PHB (P263 injection molding grade) pellets with a density of 1.3 g/cm^3^ were obtained from Biomer (Schwalbach, Germany). The renewable content is around 86.5%, and the rest is biodegradable or inert material. The material is approved for food contact according to EU regulation 1935/2004. Further property details from the manufacturer (Biomer, Germany) can be found in Appendix A Table A11.

ICPP (C7069-100NA) pellets with a density of 0.9 g/cm^3^ were obtained from Braskem Europe (Braskem, Germany). The MFR value is 100 g/10 min. The material is approved for food contact according to EU regulation 1935/2004. Further property details from the manufacturer (Braskem, Germany) can be found in Appendix A Table A12. For convenience, the abbreviation PP has been used in the following sections to refer to ICPP.

### 2.2. Processing and Mechanical Recycling

The moisture content of the received PHB was 0.18%, as tested in an FMX HydroTracer (aboni GmbH für Mess-und Automatisierungstechnik, Schwielosee, Germany). The material was predried at 60 °C for 2 h in a DP615 dryer (Piovan S.p.A, Venice, Italy) prior to processing in a co-rotating twin screw extruder ZSE18HPe-48D (Leistritz Extrusionstechnik GmbH, Nürnberg, Germany) with a screw diameter of 17.8 mm and an L/D ratio of 48 and equipped with a double-strand die.

To simulate mechanical recycling, five times repeated extrusions of the virgin material were carried out. No additives were added at any time to the two systems. The temperature profiles used for the PHB and PP systems are detailed in Table 1 (only the most important parameters are shown, and the remaining data are attached in Appendix A, Table A7 and Table A8). On extrusion, the polymer strands were air-cooled on a conveyor belt and granulated.

**Figure 1:**
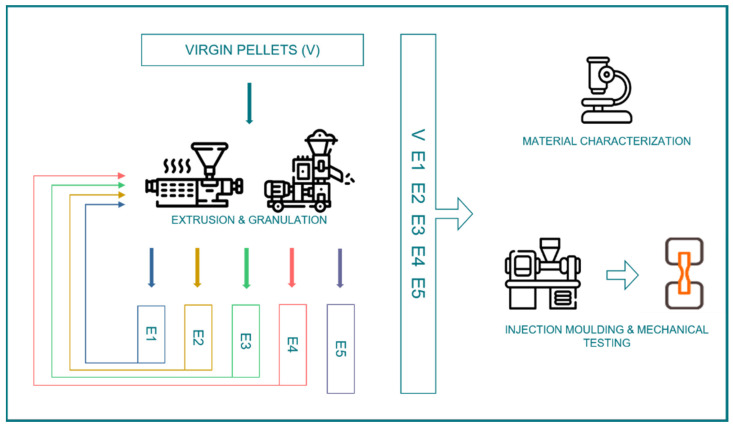
Schematic of followed mechanical recycling pathway.

Thereafter, multipurpose test specimens (ISO 3167 [53]) were fabricated on the injection molding machine ALLROUNDER 320 C (Arburg GmbH + Co. KG, Lossburg, Germany) with the processing parameters specified in Table 2 (more detailed process settings are summarized in Appendix A, Table A9 and Table A10). The variation of the injection rate and nozzle temperature for different recycling steps was necessary to obtain good parts. A schematic of the experimental design is shown in Figure 1.

### 2.3. Testing Methods

Gel permeation chromatography (GPC): Molar mass distributions (MMDs), average molar masses (*M*_n_, *M*_m_) and dispersities (Đ) for PHB samples were analyzed via GPC on a PL-GPC-50 setup equipped with an automatic autosampler, isocratic pump, two Agilent ResiPore 3 µm columns, refractive index, viscosimetry and multi-angle (15 and 90°) light scattering detectors. Tetrahydrofuran (THF) was used as an eluent at the flow rate of 1 mL min^−1^. The columns were kept at 30 °C. The calibration was performed with polystyrene standards (mass average molar mass *M*_m_ range from 6 × 10^6^ g mol^−1^ to 1.62 × 10^2^ g mol^−1^). Before the analysis, 10 mg of the sample was dissolved in 1 mL of THF and shaken at room temperature for 1 h to ensure complete dissolution. For the analysis of PP samples, an Agilent high-temperature GPC 1260 Infinity II system equipped with an autosampler, three Olexis 3µm columns, refractive index, viscosimetry and multi-angle (15 and 90°) light scattering detectors was used. The analysis was performed in trichlorobenzene at 160 °C. The columns were calibrated with polystyrene standards (mass average molar mass *M*_m_ range from 6 × 10^6^ g mol^−1^ to 1.62 × 10^2^ g mol^−1^). Before the analysis, ca. 8 mg of the sample was dissolved in 4 mL of the solvent, heated and stirred for 1 h and then filtered through a 20 μm pore filter.

Fourier transform infrared spectroscopy (FTIR): The measurements were carried out on a Bruker 2009 spectrometer (Bruker Corporation, Billerica, MA, USA) with the OPUS 6.5 software. The measurements were carried out in attenuated total reflectance (ATR) and absorbance mode. The spectral resolution was 4 cm^−1^. For each measurement, 32 scans were averaged in the region of 4000–600 cm^−1^.

Scanning electron microscopy (SEM): A Phenom Pro Desktop scanning electron microscope (Thermo Fisher Scientific Inc., Waltham, MA, USA) was used to obtain images of the surface morphology of representative impact specimens. The edge was cut out and placed under the microscope, and images were taken at around 500× magnification. The mode used was 5 kV BSD-full. The samples were sputtered with a thin Au coating prior to scanning.

Melt mass flow rate (MFR): The melt mass flow rate was measured using the melt flow tester MP1200 (Tinius Olsen, Salfords, UK). The temperature was set to 180 °C for PHB and 230 °C for PP. The load used was 2.16 kg. The measurements were replicated three times each.

Differential scanning calorimetry (DSC): The machine used was a Mettler Toledo (Greifensee, Switzerland) DSC 1 with a GC 200 gas controller and STAR^e^ evaluation software Version 16.00. The sample testing was conducted under a nitrogen atmosphere, and the pans used were 40 µL aluminum crucibles. The heating rate was 10 K/min, and the cooling rate was 20 K/min. The PHB samples were heated from 20 °C to 220 °C. The PP sample was heated from 20 °C to 260 °C. The temperature to which the PHB samples were heated was intentionally kept lower than that for PP as they are heat-sensitive. The non-isothermal runs consisted of two heating ramps and one cooling ramp. The sample mass ranged from 9 to 12 mg with three replicates of each.

The degree of crystallinity $X_{c}$ was calculated via [54]

$$X_{c}=\frac{{∆H}_{m}}{{∆H}_{m,100\%}}\cdot 100\%$$

where ${∆H}_{m,100\%}$ is the melting enthalpy of a 100% crystalline material, which is 146 J/g for PHB according to [55] and 207 J/g for PP according to [54].

Thermogravimetric analysis (TGA): A TGA/DSC 1 (Mettler Toledo, Greifensee, Switzerland) with a GC 200 gas controller was used. The measurements were conducted from 25 °C to 600 °C under a nitrogen atmosphere at 10 K/min to observe the degradation temperature. The measurement was replicated two times as the results were always identical. The mass of the sample was from 10 to 20 mg, and the pan was 70 µL aluminum oxide.

Rheology: The machine used for the PHB system was the modular compact rheometer MCR 501 (with the electrical temperature device ETD 400 Anton Paar GmbH., Graz, Austria), and for the PP system, an MCR 702 MultiDrive with a CTD 600 convection temperature device (Anton Paar GmbH., Graz, Austria) was used. The samples were compression molded into discs of 25 mm in diameter and 2 mm in height with the Collin P200PV press (Dr. Collin GmbH, Ebensberg, Germany). Initially, an amplitude sweep was conducted to determine the limits of the linear viscoelastic region (LVE) by shear deformation in the range of 0.1–100% and at the frequency of 1 rad/s. The LVE region of PHB was determined as 3% shear deformation, and that of PP was determined as 10% shear deformation. The frequency sweep measurements were performed at strains in the linear viscoelastic range in the frequency range from 0.1 rad/s to 500 rad/s with a plate–plate geometry of 25 mm diameter at a 1 mm gap in accordance with ISO 6721 [56]. The PHB samples were tested at 190 °C, and the PP at 180 °C (these temperatures were chosen based on their melting point observed by DSC measurements). The measurement was replicated three times. To avoid thermal degradation, the measurement for the frequency sweep was conducted starting from 0.1 rad/s as at low frequencies, the measurement will require more time, and this would help avoid thermal degradation in the measurement.

Tensile tests: Tensile measurements were conducted on a Zwick Z010 universal testing machine (Zwick/Roell GmbH & Co. KG, Ulm, Germany) equipped with a 10 kN load cell. The measurements were conducted according to ISO 527 [57] and replicated five times. The modulus was tested at 1 mm/min; after that, the speed of testing was 5 mm/min until break. The gauge length was 115 mm. The strain was measured by displacement transducers with a displacement of 50 mm. The tests were performed at standardized conditions (23 °C air temperature, 50% relative humidity) with ISO 527-1A specimens. For each sample, five replicate measurements were performed. The evaluation of the Young’s modulus, the strain at break and the tensile strength was performed with the software testXpert II Version 3.61 (Zwick/Roell GmbH & Co. KG, Ulm, Germany).

Impact tests: Charpy impact tests were conducted on a CEAST Resil 25 (CEAST Spa, Pianezza, Italy) impact tester equipped with a 0.5 J impact pendulum. The tests were performed according to EN ISO 179-1 [58] at standardized conditions. The specimens were notched prior to testing with a Type A notch (ISO 179-1eA). The Charpy notched impact strength was evaluated as impact energy divided by the net cross-section area. The mean and standard deviation values were determined for six to seven samples.

## 3. Results and Discussion

### 3.1. Molecular Properties

#### 3.1.1. Analysis of the Molar Mass and Molar Mass Distribution via Gel Permeation Chromatography, Melt Flow Rate and Rheology

Table 3 summarizes the changes in the mass average molar mass (*M*_m_) and dispersity (Ð) for the multiple reprocessing of PHB and PP. For both types of polymers, we observe a decrease in *M*_m_, indicating that the main mechanism of degradation is chain scission. The increase in the Ð values is also consistent with the random scission of the polymer chains. As the material had been dried before processing and reprocessing, we can assume hydrolysis is not one of the causes of the decrease in molecular weight. Our observations are in accordance with the literature reports. In particular, Beltran et al. [59] observed a decrease in *M*_m_ for virgin and recycled PLA. The weight average molecular weight is supposed to be linked more to high-molecular-weight polymer chains, whereas the number average molecular weight (*M*_n_) is more related to low-molecular-weight polymer chains. Corre et al. [60] also noticed a decrease in the molecular weight (33% reduction) and the dispersity value (Ð) for all the PHAs studied, from the raw pellets to the injection-molded samples, due to melt processing.

For PP, a decrease in *M*_m_ and *M*_n_ was also observed in the study conducted by Tochacek et al. [52,61] on ICPP for the first and fifth extrusions, due to the β-chain scission. The decrease in *M*_m_ was also investigated in [62] in which virgin PP was compared with recycled PP from industrial regrind. Alongside the decrease in the molar mass, the dispersity values increased for each recycling step (Table 4).

Consequently, both polymers exhibit chain scission during multiple reprocessing.

**Figure 2:**
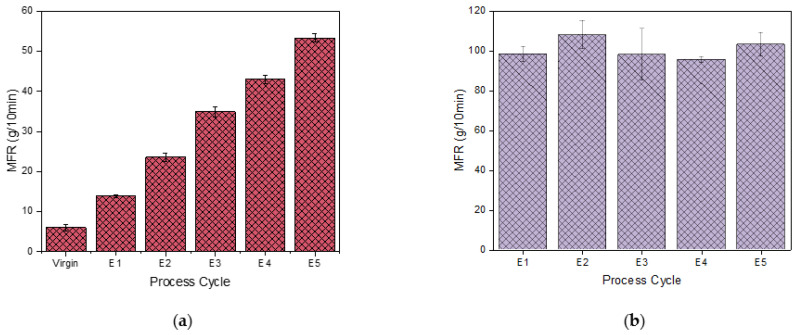
Melt flow rate (MFR) of PHB virgin–E5 samples (**a**) and of PP E1–E5 (**b**).

In Figure 2a, the melt flow rates of the PHB virgin material and of the cycles E1–E5 are illustrated. The melt flow rate increases linearly with each cycle. According to Morackzewski et al. [30], multiple processing of P(3,4HB) for ten cycles also resulted in 3 times increased MFR values from P0 to P10 due to degradation with a consecutive injection molding cycle, resulting in a molecular weight decrease. Similar to the observation by Badia et al. [63], the flowability significantly increased with the reprocessing cycle number, which is consistent with the GPC observations.

The MFR of PP from E1 to E5 is depicted in Figure 2b. It is observed that the increase in MFR is not as linear and significant as that observed in PHB, hence being more stable. However, we observed a 2 times decrease in the *M*_m_ via GPC. Thus, GPC is more sensitive to the degradation of PP than MFR. Tochacek et al. [51] observed that as the number of reprocessing cycles progresses, the MFR increases for three types of optimized PP systems. Guo et al. [64] also stated that a decrease in molecular weight is reflected in an increase in MFR as the cycles progress for multiple recycling of PP. This was also validated by two additional publications [61,65]. Consequently, the insignificant decrease in MFR reported here can be attributed to the significant experimental error and lack of sensitivity.

For an in-depth study of the flow behavior of the virgin and reprocessed samples, we performed rheological measurements.

**Figure 3:**
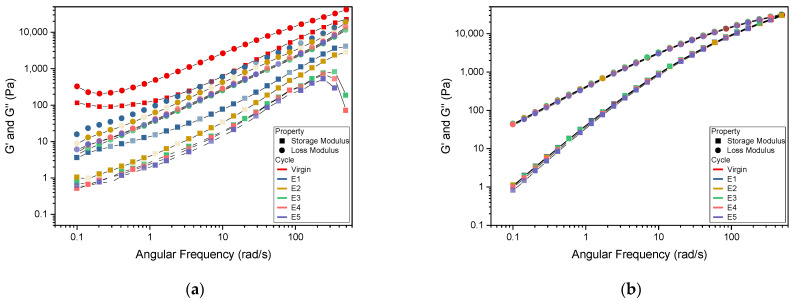
Frequency sweep curves for (**a**) PHB and (**b**) PP.

In the frequency sweep graph, the loss modulus G” curves were above the storage modulus G’ curves for both the PHB and PP series (Figure 3a,b). This means the energy dissipation due to viscosity is larger than the elastic energy stored [66]. There was a distinct difference noticed between the virgin PHB modulus curve and the E1 series (Figure 3a). E2–E5 decrease slightly in their G” curves and are almost overlapping in the storage modulus depiction. At higher frequencies, there is a drop observed in the G’ curves of E3–E5 which could not be explained. It could be that above 200 rad/s we are going above the limit of the setup for E3–E5. In the study by Park et al. [66], the G” curve was higher than the G’ curve and rising steadily, similar to the curves obtained in this study.

This is a clear difference from the PP series, in which the modulus curves overlap from virgin to E5 with a cross-over point probably achieved at a frequency > 500 rad/s (Figure 3b).

**Figure 4:**
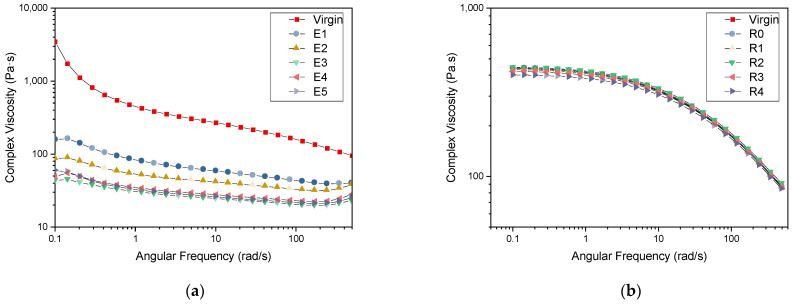
Complex viscosity vs. angular frequency curves for (**a**) PHB and (**b**) PP.

In the complex viscosity vs. angular frequency curves (Figure 4a,b), a clear decrease is observed from the curves of virgin to E1 followed by a slight decrease to E2. The curves of E3–E5 are overlapping each other. This is in keeping with the results observed for the MFR values for the PHB series. Except for the virgin PHB, all the samples exhibit a non-Newtonian flow profile, with a decrease in the viscosity profile as the frequency increases [60].

It should be noted that the selected grade of PP exhibits rather low molar mass (*M*_n_ = 6.4 × 10^4^ g/mol) and high MFR (100 g/10 min). Upon degradation, the molar mass decreases even more, making it difficult to detect the difference in the flow properties. Consequently, we observe nearly no changes in MFR and rheological measurement. However, the GPC results indicate a 10 times decrease in the *M*_n_ value, making it more sensitive to the changes in the molar mass for low-molar-mass polymers.

#### 3.1.2. Analysis of the Functional Group Evolution via Fourier Transform Infrared Spectroscopy

**Figure 5:**
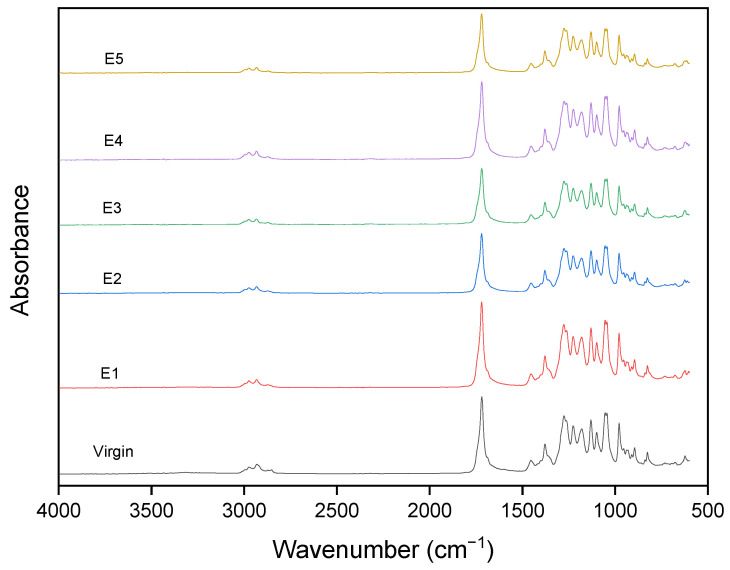
FTIR spectra of PHB after different reprocessing cycles.

In Figure 5, which refers to the spectra of the PHB series, the strongest peak observed was C=O carbonyl stretching at 1722 cm^−1^. C-C-O stretches were observed at 1224 and 1179 cm^−1^. O-C-C- was observed at 1095 and 1050 cm^−1^. The CH3 asymmetric stretch was observed at 2974 cm^−1^, and the CH2 asymmetric stretch at 2931 cm^−1^. The CH3 symmetric stretch was observed at 2867 cm^−1^. The CH3 symmetric bend (umbrella mode) was observed at 1375 cm^−1^. No new formation of groups was noticed even after five processing cycles.

**Figure 6:**
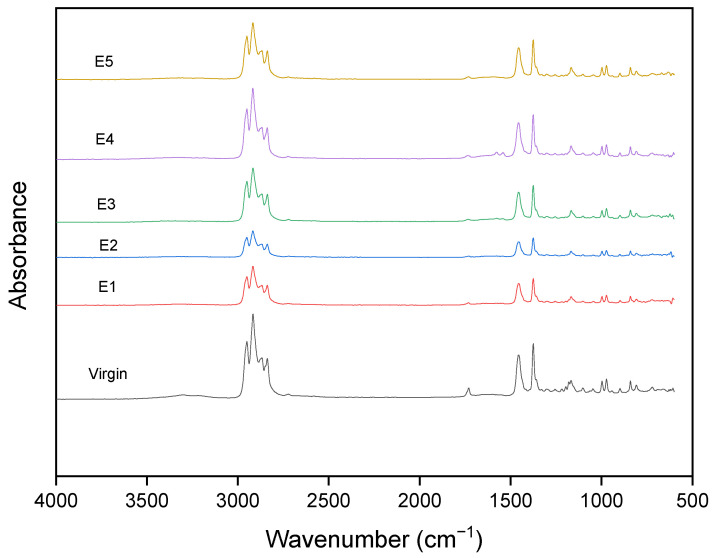
FTIR spectra of PP after different reprocessing cycles.

For the PP series, the strongest peaks naturally occur in the region of 3000–2800 cm^−1^ with four peaks referring to the CH3 and CH2 stretches due to both methylene and methyl groups present in PP (Figure 6). The peak at 1373 cm^−1^ refers to CH3 umbrella mode, and the small peak at 724 cm^−1^ refers to CH2 rock because of the ethylene contribution to the copolymer. A small peak is visible at 2723 cm^−1^ which is a characteristic peak of PP [67]. A broad, flat peak was observed between 3500 and 3000 cm^−1^. This could be due to hydroxyl groups or hydrogen bonds in impurities, and the peak at 3304 cm^−1^ could be due to an amide stretch vibration. There is a visible peak with reference to C=O carbonyl stretching at 1733 cm^−1^ which could be due to thermo-oxidative degradation/photodegradation or due to some additive present made of esters [67,68]. Martinez Jothar et al. [69] studied the carbonyl band as it has an almost constant position and is a good indicator of degradation in the PP system. Wang et al. [70] discussed the possibility of the origin of the carbonyl group most probably from an antioxidant present in the PP. Moreover, in their study, the peak was partially reduced with six recycling cycles, leading to no difference in the graphs of the virgin and recycled material. This was observed in our study as well with five reprocessing cycles. This is further confirmed by the study conducted by Guerrica-Echevarria et al. [71] who proved that the peak occurring in the carbonyl range was due to the stabilizer they added. According to Guo et al. [64], after 15 extrusions of PP were conducted, the ester peak at 1740 cm^−1^ was indicative of oxidative degradation. In our study, the peak belongs to the stabilizer/antioxidant as it is consumed after the first reprocessing cycle.

### 3.2. Morphological Properties: Analysis of the Surface Morphology via Scanning Electron Microscopy

**Figure 7:**
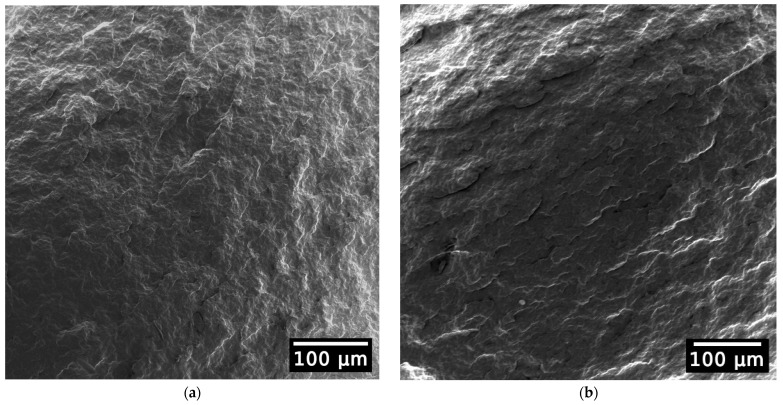
SEM images of PHB-virgin sample (**a**) and PHB-E5 sample (**b**).

SEM images help to characterize the surface morphology of the material tested. The PHB samples of virgin (Figure 7a) and E5 (Figure 7b) material look almost identical, with a slight increase in surface roughness similar to that found in the multiple reprocessing of PHBV by Zaverl et al. [28]. This is in contrast to the multiple reprocessing of PLA in which distinct microcracks were visible in the sixth cycle and were correlated with the impact values as described by Agüero et al. [72].

**Figure 8:**
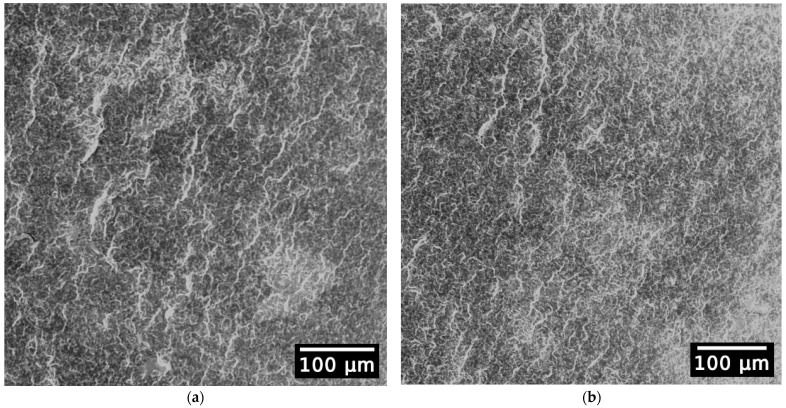
SEM images of PP-virgin sample (**a**) and PP-E5 sample (**b**).

In the case of PP, the images (Figure 8a,b) are almost identical too, as also reported by Tochacek et al. [47,52], who under increased magnification and after extraction with heptane were able to compare the size of the rubbery phase and noted there was no decrease in particle size with multiple processing. This observation by Tochacek et al. was in contradiction to the work performed by Alotaibi et al. [49] where the ethylene propylene rubber (EPR) of heterophasic PP was visible and, with each reprocessing step, the EPR steadily decreased in size, which is also similar to the observations of Bouaziz et al. [73].

In summary, the decrease in the polymers’ molar mass observed via GPC did not cause significant changes in the surface morphology.

### 3.3. Thermal Properties: Analysis via Differential Scanning Calorimetry and Thermogravimetric Analysis

**Figure 9:**
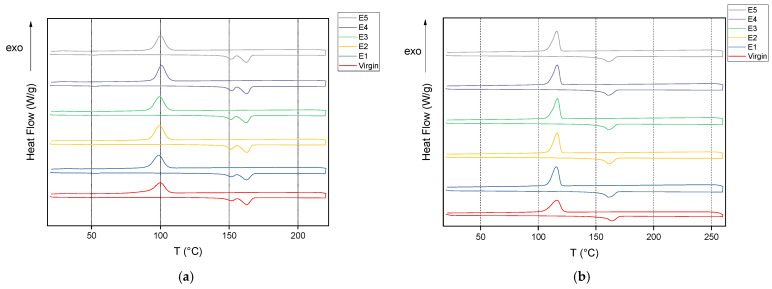
Representative DSC curves for the 2nd heating and cooling cycle of (**a**) PHB virgin–E5 and (**b**) PP virgin–E5.

The second heating scan and the cooling scan are shown in Figure 9. The first heating scan shows a single melting peak at 177 °C, and the second heating scan shows twin melting peaks with the major peak at 162 °C (Table 5).

In the first heating scan, the enthalpy of melting increases with each process cycle, starting from 58.6 J/g for the virgin material to 69.7 J/g for E5 as in Table 5. Additionally, as the processing cycles progress, the crystallinity increases from virgin to E1, and then it remains mostly constant. This is in correlation with the study of Rivas et al. [27] who also noticed an increase in the crystallinity as the cycles progress. This is associated with the decrease in the molecular weight with the number of extrusion cycles as was explained earlier in the GPC section. This decrease enhances the mobility of the molecules, which enhances the crystallization process. The melting temperature $T_{m}$ did not vary a lot with each cycle [28] and was approx. 177 °C in the first heating cycle.

Polypropylene shows an initial decrease in the $T_{m}$ of approx. 1 °C from the virgin material which is then held constant as the cycles progress (Table 6). The degree of crystallinity was similar throughout except for E3 in which there is a drop observed. This is in line with the results observed by [71] and also explained in [74] that the degradation causes enhanced mobility of the chain segments.

The cooling scans as shown in Figure 9a,b in the cases of PHB and PP both show a single crystallization peak. The increase in crystallization enthalpy ${∆H}_{c}$ for PHB and PP also points to chain scission occurring as the extrusion cycles progress (Table 5 and Table 6).

In the second heating scan, which is the material behavior shown after erasure of the previous thermal history, the presence of two melting peaks is observed (Figure 9a) for all the cycles of PHB, and the value of the main melting peak decreases only marginally from 162.9 to 162.6 °C, making it remarkably steady in spite of the increase in the cycles. There is also a slight melting peak detected around 50 °C in the PHB series which could be due to the presence of an additive [60]. The degree of crystallinity was also calculated from the melting enthalpy of the second heating scan. The melting point (162.9 °C), melting enthalpy (68.5 J/g) and degree of crystallization (46.9%) are similar to those of the additivized PHB studied in [75].

For PP, the second heating scan only shows a single melting peak (Figure 9b) which is at 163 °C for the virgin PP and then decreases to 161 °C in cycle E1 and stays at that value for the subsequent cycles (Table 6). Overall, PHB seems to be steadier in the melting properties in comparison to PP.

In summary, the degree of crystallinity is overall higher for PHB compared to PP, which will be explored further in Section 3.4 on material properties.

**Figure 10:**
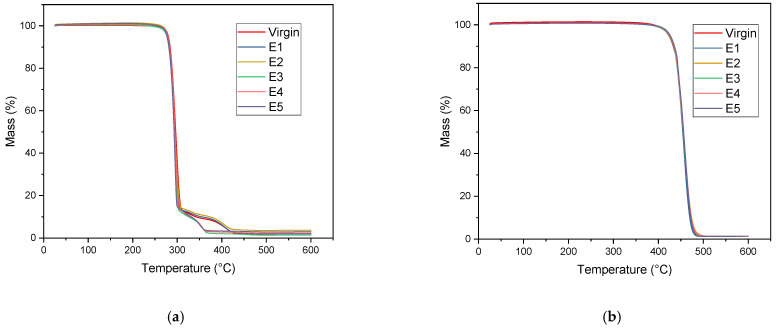
Representative TGA curves of (**a**) PHB and (**b**) PP for all processing cycles.

Figure 10a shows the mass loss of PHB over cycles 1–5 and the virgin material. As is evident from the graph, two types of two-step degradation were observed, one with virgin, E1 and E2 and the other with E3, E4 and E5, and additionally, a low level of residue at 600 °C remained in all cycles. A degradation temperature change was observed from the virgin material to the E5 cycle to a maximum of around 2 °C. The residue could relate to the presence of plasticizers and/or other additives as observed in a study by Corre et al. [60]. Weinmann et al. [76] obtained similar curves with the addition of plasticizers and other additives to the pure PHB powder.

The thermal stability of PHB was assured by only marginal changes (even a slight increase) in the degradation temperature obtained by TGA in the five processing cycles as seen in Table 7. Moraczewski [30] observed a similar result and stated that as the degree of crystallinity increases (as observed from the DSC), so does the thermal stability.

Figure 10b shows the degradation pattern for PP. It is a single-step degradation representative of random chain scission, but in our study, the degradation temperature is in a range from 435.3 to 438 °C (summarized in Table 8), differing slightly from the results of others who observed a decrease in the degradation temperature of reprocessed PP in comparison to the virgin polymer [65,70,77]. The results demonstrate the good thermal stability of the recycled PP.

### 3.4. Material Properties: Analysis via Tensile and Impact Testing

**Figure 11:**
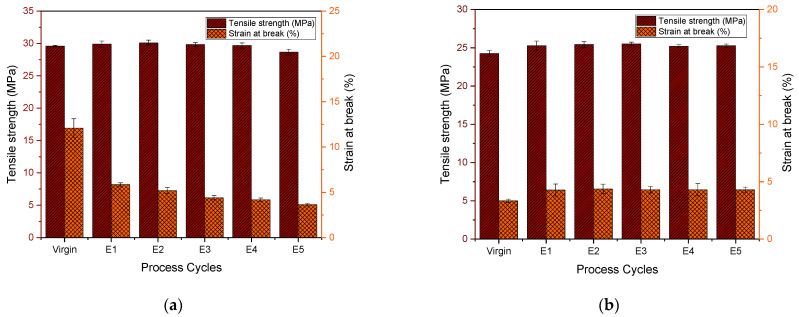
Tensile strength and strain at break of (**a**) PHB and (**b**) PP for virgin material and after 1–5 reprocessing cycles (E1–E5).

Figure 11a shows the tensile strength of virgin and reprocessed PHB. It should be noted that from virgin to E4 we observe statistically similar values, and only E5 is reduced. The decrease in the tensile strength is consistent with the decrease in the molar mass observed above. However, our results differ from the study by Rivas et al. [27] who noticed a strong decrease from virgin to E3 with more than 50% reduction in tensile strength. Nevertheless, these authors did not make a link to the changes in the molecular properties. Evidently, we can conclude that the decrease in the molar mass *M*_m_ up to 2 times causes an insignificant decrease in the tensile strength.

The tensile stress–strain curve of virgin PHB is type 3, while the curves of E1–E5 are type 1 according to ISO 527. In this, a transition from a tough material to a brittle material is evident. El-Taweel et al. [78] related the tensile strength values to the degree of crystallinity in PHB polymers, and above a 40% degree of crystallinity, they are said to be brittle, which explains the findings obtained here as well. This is in fact similar to the study by Zaverl et al. [28] regarding the recycling of PHBV, and in that study too, there was a decrease observed only after the fifth extrusion. The dependence of tensile strength on *M*_n_ as illustrated in [79] can be observed in our findings as well: in E2, the *M*_n_ drops more, which leads to a decrease in the number of nuclei for crystallization and hence a lower number of spherulites with increased diameter which leads to brittleness.

**Figure 12:**
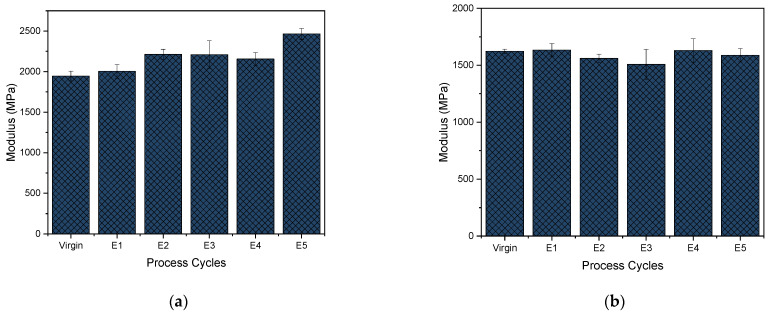
Young’s modulus of (**a**) PHB and (**b**) PP for virgin material and after 1–5 reprocessing cycles (E1–E5).

Figure 11a shows a statistical difference between virgin and E1 PHB strain-at-break values. After that, they remained similar throughout. This is in keeping with the fact that the degree of crystallinity is above 40% [28]. Pillin et al. [80] discussed the strain-at-break drop due to a decrease in chain length and an increase in crystallinity. The Young’s modulus of PHB (Figure 12a) slightly increases as the cycles progress, which means that it is progressively becoming stiffer. This could be due to the decrease in molecular weight being balanced by an increase in crystallinity, similar to a study performed with PLA recycling [80].

In Figure 11b, the tensile strength of the virgin PP is statistically different from that of E1 PP. Thereafter, from E1 to E5, the values are statistically similar. All the curves obtained are characteristic for a brittle material behavior, and the strain at break follows the same pattern with not much difference between the cycles. This is in contrast to the reprocessing performed by La Mantia et al. [81], in which they did not notice this increase. There, the values of tensile strength and strain at break all dropped from the virgin polymer onwards. The Young’s modulus (in Figure 12b), on the other hand, showed the same trend of first decreasing and then increasing as that of La Mantia et al. [81] due to the dual phenomenon of a reduction in molecular weight followed by an increase in crystallinity.

In summary, all the tensile properties, tensile strength, strain at break and Young’s modulus, of PHB are higher than those of the PP tested due to the higher degree of crystallinity observed as explained above. The transition of virgin PHB (tough) to E1–E5 (brittle) has also led to a decrease in strain-at-break values. But as PP was brittle throughout, it had similar low values of elongation at break. The increase in Young’s modulus with reprocessing is due to the decrease in molar mass balanced by the increase in the degree of crystallinity.

**Figure 13:**
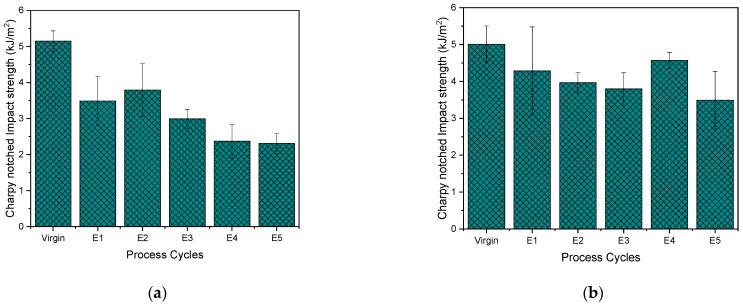
Charpy notched impact strength of (**a**) PHB and (**b**) PP for virgin material and after 1–5 reprocessing cycles (E1–E5).

The Charpy notched impact strength of PHB supports the strain-at-break results by revealing the progressive brittleness of the samples with a 50% reduction at the end of E5 (Figure 13a). This reflects the investigations of Zenckiewicz et al., Bruster et al. and Badia et al. [82,83,84] in which a decrease in the impact values of multi-extruded PLA was noticed. The largest decrease in this study was noticed from virgin to E1 to a value of 3.485 kJ/m^2^, around 67% of that of the virgin material, which was also proven by Tukey ANOVA to be statistically different. This is in contrast to the study of Zaverl et al. [28], in which there was in fact a slight increase observed in the Izod impact strength on repeated extrusions of PHBV.

The Charpy notched impact strength of PP mirrors that of reprocessed ABS in the study by Rahimi et al. [85] (Figure 13b). Similar to this study, the Izod values decreased with each reprocessing step as well, except for E4 where there was a slight increase in the Charpy values, again followed by a decrease in E5. But according to Tukey, the differences are not so significant as an overlap is noticed for E4 with E3 and E5. The decrease in the Izod impact strength values of a PP copolymer in the study by Alotaibi et al. [49] for cycles from E1 to E3 was similar to the trend observed here.

In summary, a decrease in notched Charpy impact strength is observed in both PHB and PP with reprocessing due to chain scission.

## 4. Conclusions

The demand for alternative feedstocks is of utmost importance for activities aimed at a circular economy. The current end-of-life scenarios for biobased and biodegradable plastics are geared towards industrial composting. The mechanical recyclability of polyhydroxybutyrate (PHB) has seldom been of interest.

In this regard, this study was conducted to check the feasibility of industrial reprocessing of PHB. Polypropylene (PP) was studied as a reference, with each material being reprocessed five times.

- For both materials, a decrease in molecular weight was observed in gel permeation chromatography, which can be attributed to chain scission occurring on repeated processing. There was a 57% decrease in *M*_m_ from virgin to E5 PHB, and in PP, there was a 65% decrease from virgin to E5.
- However, Fourier transform infrared spectroscopy revealed that no new groups were formed.
- Sufficient thermal stability over the cycles was observed as the degradation temperature stayed almost constant. An increase in crystallinity was observed by differential scanning calorimetry which translated to increased Young’s modulus (stiffness) over the cycles due to a reduction in molecular weight being balanced by the increase in crystallinity. E5 PHB has 19.2% higher crystallinity than virgin PHB and correspondingly 26.7% higher Young’s modulus than virgin PHB.
- The tensile strength of PHB was overall 20% higher than that of PP, and a statistical difference was only observed after five reprocessing cycles for PHB.
- The viscosity decrease evaluated for PHB by MFR on reprocessing was corroborated further by frequency sweep studies in contrast to the PP series, where the viscosity remained remarkably steady over the reprocessing cycles.
- On the other hand, the strain at break of virgin PHB was 4 times higher than that of PP, and it decreased significantly during the first reprocessing cycle and then remained similar.
- The Charpy notched impact strength showed a similar trend for both materials.

Further modifications and studies are needed to check for the improvement in rheological, strain-at-break and impact properties with the addition of virgin PHB and/or other additives. Nevertheless, this study shows the potential for PHB to be mechanically recycled as is done with conventional polypropylene.

## Figures and Tables

**Table 1:** Extrusion parameters for PHB and PP.

Material	Throughput Rate (kg/h)	Die Temperature (°C)	Screw Rotation Speed (rpm)
PHB	5	160	500
PP	4	190	300

**Table 2:** Injection molding processing parameters.

Material	Injection Rate (cm^3^/s)	Nozzle Temperature (°C)	Mold Temperature (°C)	Cooling Time (s)
PHB	10 (V) 15 (E1–E5)	185 (V) 165 (E1–E5)	38	30
PP	25	180 (V and E1) 190 (E2–E5)	30	35

**Table 3:** Molecular weight change of PHB with processing cycles.

Sample	*M*_m_ (g/mol)	*M*_n_ (g/mol)	Ð (-)
Virgin	5.4 × 10^5^	2.9 × 10^5^	1.87
E1	3.2 × 10^5^	1.9 × 10^5^	1.71
E2	2.4 × 10^5^	1.3 × 10^5^	1.79
E3	2.2 × 10^5^	1.2 × 10^5^	1.78
E4	2.2 × 10^5^	1.4 × 10^5^	1.61
E5	2.3 × 10^5^	1.2 × 10^5^	1.97

**Table 4:** Molecular weight change of PP with processing cycles.

Sample	*M*_m_ (g/mol)	*M*_n_ (g/mol)	Ð (−)
Virgin	1.8 × 10^5^	6.4 × 10^4^	2.7
E1	1.3 × 10^5^	1.6 × 10^4^	8.3
E2	9.8 × 10^4^	1.2 × 10^4^	7.9
E3	8.8 × 10^4^	1.04 × 10^4^	8.4
E4	7.8 × 10^4^	5.6 × 10^3^	13.8
E5	6.3 × 10^4^	6.2 × 10^3^	10.1

**Table 5:** Thermal properties of PHB as determined by DSC.

Process Cycle	1st Heating Scan			Cooling Scan		2nd Heating Scan		
PHB	T_m_	ΔH_m_	X_c_	T_c_	ΔH_c_	T_m2_ ^1^	ΔH_m_	X_c_
	(°C)	(J/g)	(%)	(°C)	(J/g)	(°C)	(J/g)	(%)
Virgin	176.4 ± 0.5	58.6 ± 0.6	40.1 ± 0.4	100.3 ± 0.5	54.2 ± 1.1	162.9 ± 0.1	68.5 ± 0.7	46.9 ± 0.5
E1	177.0 ± 1.0	66.1 ± 1.2	45.3 ± 0.9	98.6 ± 0.2	58.9 ± 0.7	162.9 ± 0.2	68.1 ± 0.3	46.6 ± 0.2
E2	176.7 ± 0.3	64.2 ± 0.8	44.0 ± 0.6	99.5 ± 0.4	62.0 ± 0.8	162.9 ± 0.1	69.1 ± 1.5	47.3 ± 1.1
E3	175.8 ± 0.4	67.6 ± 0.5	46.3 ± 0.4	100.0 ± 0.7	62.3 ± 0.9	162.7 ± 0.3	65.4 ± 1.5	44.8 ± 1.1
E4	176.4 ± 0.2	68.3 ± 0.7	46.8 ± 0.5	100.5 ± 0.6	62.4 ± 0.5	162.6 ± 0.1	68.8 ± 1.8	47.1 ± 1.3
E5	176.0 ± 0.4	69.7 ± 1.5	47.8 ± 1.0	100.5 ± 0.3	62.4 ± 1.3	162.6 ± 0.1	68.2 ± 2.1	46.7 ± 1.4

^1^ Refers to the major melting peak in the twin peaks.

**Table 6:** Thermal properties of PP as determined by DSC.

Process Cycle	1st Heating Scan			Cooling Scan		2nd Heating Scan		
PP	T_m_	ΔH_m_	X_c_	T_c_	ΔH_c_	T_m2_	ΔH_m_	X_c_
	(°C)	(J/g)	(%)	(°C)	(J/g)	(°C)	(J/g)	(%)
Virgin	169.8 ± 1.0	73.3 ± 1.3	35.4 ± 0.6	115.5 ± 0.8	81.1 ± 5.4	163.1 ± 0.6	71.3 ± 4.1	34.4 ± 2.0
E1	168.8 ± 0.3	76.4 ± 2.7	36.9 ± 1.3	116.1 ± 0.2	84.8 ± 1.1	160.8 ± 0.3	78.0 ± 1.0	37.7 ± 0.5
E2	168.8 ± 0.5	76.7 ± 0.8	37.1 ± 0.4	116.6 ± 0.04	84.6 ± 0.8	161.2 ± 0.2	73.3 ± 0.6	35.4 ± 0.3
E3	168.8 ± 0.4	67.5 ± 1.4	32.6 ± 0.7	116.7 ± 0.2	84.5 ± 0.9	160.8 ± 0.1	75.1 ± 0.9	36.3 ± 0.4
E4	168.9 ± 0.4	73.9 ± 1.7	35.7 ± 0.8	116.3 ± 0.1	84.5 ± 0.9	160.5 ± 0.2	76.7 ± 0.4	37.0 ± 0.2
E5	168.8 ± 0.1	76.0 ± 0.9	36.7 ± 0.4	116.4 ± 0.1	84.2 ± 0.1	160.5 ± 0.2	75.0 ± 0.9	36.2 ± 0.4

**Table 7:** Degradation temperature (Td) of PHB with processing cycle.

Sample	T_d_ (°C)
Virgin	284.2 ± 0.6
E1	284.6 ± 0.6
E2	284.7 ± 0.6
E3	286.0 ± 0.4
E4	286.2 ± 0.1
E5	286.8 ± 0.1

**Table 8:** Degradation temperature (Td) of PP with processing cycle.

Sample	T_d_ (°C)
Virgin	435.3 ± 1.4
E1	438.0 ± 0.8
E2	435.7 ± 0.6
E3	436.4 ± 0.4
E4	437.1 ± 0.2
E5	437.2 ± 0.9

## Data Availability

The data presented in this study is available upon request from the corresponding author.

## Appendix A

**Table A1:** PHB tensile strength. Grouping information using the Tukey method and 95% confidence.

Factor	N	Mean	Grouping	
E2	5	30.1	A	
E1	5	29.9	A	
E3	5	29.9	A	
E4	5	29.7	A	
Virgin	5	29.6	A	
E5	5	28.7		B

Means that do not share a letter are significantly different.

**Table A2:** PHB strain at break. Grouping information using the Tukey method and 95% confidence.

Factor	N	Mean	Grouping			
Virgin	5	12.1	A			
E1	5	5.9		B		
E2	5	5.2		B	C	
E3	5	4.4			C	D
E4	5	4.2				D
E5	5	3.7				D

Means that do not share a letter are significantly different.

**Table A3:** PP tensile strength. Grouping information using the Tukey method and 95% confidence.

Factor	N	Mean	Grouping	
E3	5	25.5	A	
E2	6	25.4	A	
E1	6	25.3	A	
E5	5	25.3	A	
E4	5	25.2	A	
Virgin	5	24.3		B

Means that do not share a letter are significantly different.

**Table A4:** PP strain at break. Grouping information using the Tukey method and 95% confidence.

Factor	N	Mean	Grouping	
E2	6	4.4	A	
E4	5	4.3	A	
E3	5	4.3	A	
E5	5	4.3	A	
E1	6	4.3	A	
Virgin	5	3.3		B

Means that do not share a letter are significantly different.

**Table A5:** PHB impact strength. Grouping information using the Tukey method and 95% confidence.

Category	N	Mean	Grouping		
Virgin	6	5.1	A		
E2	6	3.8		B	
E1	6	3.5		B	
E3	6	3.0		B	C
E4	6	2.4			C
E5	6	2.3			C

Means that do not share a letter are significantly different.

**Table A6:** PP impact strength. Grouping information using the Tukey method and 95% confidence.

Category	N	Mean	Grouping	
Virgin	6	5.0	A	
E4	6	4.6	A	B
E1	7	4.3	A	B
E2	7	4.0	A	B
E3	6	3.8		B
E5	6	3.5		B

Means that do not share a letter are significantly different.

**Table A7:** Recycling parameters for PHB.

Material	PHB (V-E5)
Speed (rpm)	500
T1 (°C)	50
T2 (°C)	100
T3 (°C)	160
T4 (°C)	180
T5 (°C)	180
T6 (°C)	180
T7 (°C)	170
T8 (°C)	170
T9 (°C)	160
T10 (°C)	160
T11 (°C)	160
T12 (°C)	160
TDie (°C)	160

**Table A8:** Recycling parameters for PP.

Material	PP (V-E5)
Speed (rpm)	300
T1 (°C)	25
T2 (°C)	100
T3 (°C)	170
T4 (°C)	170
T5 (°C)	175
T6 (°C)	175
T7 (°C)	180
T8 (°C)	180
T9 (°C)	180
T10 (°C)	180
T11 (°C)	180
T12 (°C)	180
TDie (°C)	190

**Table A9:** Injection molding parameters for PHB.

Parameter		PHB	
		Virgin	E1–E5
Cylinder temperature (°C)	Hopper	35	35
	C1	165	185
	C2	170	180
	C3	175	175
	C4	180	170
	Nozzle	185	165
Mold temperature (°C)		38	38
Injection speed (cm^3^/s)		10	15
Back pressure (bar)		30	30
Injection pressure (bar)		760	1032
Holding pressure (bar)		960	900
Holding pressure time (s)		9	9
Holding pressure switchover point (cm^3^)		13	12
Residual melt cushion (cm^3^)		11.5	11.3
Rest cooling time (s)		30	30
Cycle time (s)		45.81	45.14

**Table A10:** Injection molding parameters for PP.

Parameter		PP	
		Virgin, E1	E2–E5
Cylinder temperature (°C)	Hopper	40	40
	C1	150	170
	C2	160	175
	C3	170	180
	C4	180	185
	Nozzle	190	190
Mold temperature (°C)		30	30
Injection speed (cm^3^/s)		25	25
Back pressure (bar)		20	20
Injection pressure (bar)		409–330	330–360
Holding pressure (bar)		330	330
Holding pressure time (s)		5	6
Holding pressure switchover point (cm^3^)		13.8	13.8
Residual melt cushion (cm^3^)		12.58	12.58
Rest cooling time (s)		35	35
Cycle time (s)		45.01	45.01

**Table A11:** Mechanical properties of Biomer^®^ P263—data of Biomer—www.biomer.de (accessed on 4 March 2021). The mechanical tests were conducted at least 4 weeks after preparing test samples.

Type	Biomer^®^ P263
Modulus (MPa) (1 mm/min)	1730–1760
Tensile strength (MPa) (50 mm/min)	28–29
Elongation (%) (50 mm/min)	5.4
Notched impact strength 23 °C (ISO 179/1eA)	2.5
Density (g/cm^3^)	1.3
Shrinkage (%)	1.2–1.3

**Table A12:** Typical properties of Braskem C7069-100NA.

Feature	Value
Melt Flow Rate (g/10 min)	100
Density (g/cm^3^)	0.9
Flexural Modulus (MPa) *	1500
Tensile Strength at Yield (MPa) *	28
Tensile Elongation at Yield (%) *	5
Notched Charpy Impact Strength at 23 °C (kJ/m^2^) *	4

* Injection-molded specimen according to ISO 294 [86].
